# Phenologic variation of major triterpenoids in regular and white *Antrodia cinnamomea*

**DOI:** 10.1186/s40529-016-0148-4

**Published:** 2016-10-25

**Authors:** Wei-Lun Chen, Yen-Peng Ho, Jyh-Ching Chou

**Affiliations:** 1 Department of Natural Resources and Environmental Studies, National Dong Hwa University, Shou-feng, Hualien, 97401 Taiwan; 2 Department of Chemistry, National Dong Hwa University, Shou-feng, Hualien, 97401 Taiwan

**Keywords:** *Antrodia cinnamomea*, HPLC, Medicinal fungus, MS, TLC, Triterpenoid

## Abstract

**Background:**

*Antrodia cinnamomea* and its host *Cinnamomum kanehirae* are both endemic species unique to Taiwan. Many studies have confirmed that *A. cinnamomea* is rich in polysaccharides and triterpenoids that may carry medicinal effects in anti-cancer, anti-inflammation, anti-hypertension, and anti-oxidation. Therefore it is of interest to study the chemical variation of regular orange-red strains and white strains, which included naturally occurring and blue-light induced white *A. cinnamomea*.

**Results:**

The chemical profiles of *A. cinnamomea* extracts at different growth stages were compared using thin layer chromatography (TLC) and high performance liquid chromatography (HPLC). The TLC and HPLC profiles indicated that specific triterpenoids varied between white and regular strains. Moreover, the compounds of blue-light induced white strain were similar to those of naturally occurring white strain but retained specific chemical characteristics in more polar region of the HPLC chromatogram of regular strain.

**Conclusions:**

Blue-light radiation could change color of the regular *A. cinnamomea* from orange–red to white by changing its secondary metabolism and growth condition. Naturally occurring white strain did not show a significantly different composition of triterpenoid profiles up to eight weeks old when compared with the triterpenoid profiles of the regular strain at the same age. The ergostane-type triterpenoids were found existing in both young mycelia and old mycelia with fruiting body in artificial agar-plate medium culture, suggesting a more diversified biosynthetic pathway in artificial agar-plate culture rather than wild or submerged culture.

## Background

*Antrodia cinnamomea* is an expensive medicinal fungus. In natural environment, *A. cinnamomea* grows only inside *Cinnamomum kanehirae* rotten trunk which is the only known host of *A. cinnamomea*. Studies had shown that both *A. cinnamomea* mycelium and fruiting body extracts carried medicinal effects such as anti-cancer (Chen and Yang 1995), anti-inflammation (Hseu et al. 2005), anti-hypertension (Wang et al. 2003) and anti-oxidation (Hseu et al. 2002; Song and Yen 2002).

It is generally believed that the medicinal effects of *A. cinnamomea* come from its rich contents of polysaccharides and triterpenoids (Shen et al. 1997). Unique triterpenoids have been reported from *A. cinnamomea* fruiting body specifically (Geethangili and Tzeng 2011). Due to the medicinal effects of *A. cinnamomea* fruiting bodies and their rarity, the price in the market remains high. Furthermore, the high demand in obtaining fruiting bodies of *A. cinnamomea* has promoted illegal poaching of *C. kanehirae*. The indiscriminate felling of *C. kanehirae* for *A. cinnamomea* has already endangered the tree species.

Fruiting bodies of *A. cinnamomea* are yellow-orange to red-brown in most cases. However, a white variant also occurs in natural environments. The white variant is relatively rare and more expensive than regular form. It is hard to obtain white fruiting bodies of *A. cinnamomea,* and no study had been done regarding its morphology, physiology, or biochemistry. Thus, studies comparing the regular form and the white variant of *A. cinnamomea* should be conducted.

Previous studies have pointed out that light radiation can influence fungal growth, asexual or sexual reproduction, and pigment precipitation (De Fabo et al. 1976; Haggblom and Unestam 1979; Idnurm and Heitman 2005). Thus, we changed *A. cinnamomea* pigmentation by using different light treatments prompting *A. cinnamomea* to different growth variations. The red light could cause *A. cinnamomea* to generate fruiting bodies with an irregular morphology (unpublished data), whereas the blue light can whiten *A. cinnamomea*. We analyzed variations of several major triterpenoid compounds in *A. cinnamomea* and found that the compound profiles from the whitened *A. cinnamomea* were similar to those of a naturally occurring white variant of *A. cinnamomea* but reserved some characteristic peaks of the regular form of *A. cinnamomea*.

## Methods

### Fungal strains and chemicals

The *A. cinnamomea* strain B (Chu et al. 2010) was isolated from rotten *C. kanehirae* trunks containing an *A. cinnamomea* fruiting body denoted as the regular form. The *A. cinnamomea* strain G was provided by a local *A. cinnamomea* farmer denoted as the naturally occurring white variant of *A. cinnamomea*. The whitened variant of *A. cinnamomea* was induced through a 100 lm radiation of 470 nm LED light source on *A. cinnamomea* strain B. All the chemicals used were of analytical grade or higher.

### Growth of *A. cinnamomea*

The *A. cinnamomea* strains were cultured on 50% malt extract agar (MEA) medium formulated by Chu et al. (2010), which contained 10 g glucose, 10 g malt extract, 0.5 g peptone and 20 g microbiological grade agar (Becton, Dickinson and Company, Maryland, United States) in a 1 L medium. A 2 × 2 mm agar blot of *A. cinnamomea* mycelium was placed on a 90 × 20 mm disposable petri dish containing 50 ml of 50% MEA medium. Strains B (regular) and G (naturally occurring white) were grown at room temperature under dark environment. Another strain B was grown at room temperature but under a 100 lm radiation of 470 nm LED light source for blue light radiation treatment.

### Sample preparation and extraction

The samples of *A. cinnamomea* were harvested at 2-, 4-, 6-, and 8-week old, respectively. Samples were freeze-dried and grounded to powder with liquid nitrogen. A 50 mg of sample powder was extracted with 1 ml of ethyl acetate by vortexing for 1 min. The samples were then centrifuged at 13,000 rpm for 1 min to remove the residues, and the supernatants were subjected to syringe filtration (Millex-GN 0.20 µm Nylon 13 mm, Millipore Corporation, Massachusetts, United States). After filtration, the samples were air-dried with nitrogen and redissolved in 1 ml acetonitrile prior to TLC and HPLC analysis.

### TLC analysis of *A. cinnamomea* extracts

For TLC analysis, 20 μl of *A. cinnamomea* extracts in acetonitrile were applied to 5 × 10 cm TLC silica gel 60 aluminum sheets (Merck KGaA, Darmstadt, Germany). The TLC sheets were developed in a TLC developing tank with a solvent system of chloroform: methanol: water = 85:14:1 (v/v/v) (Ehmann 1977). After TLC developing was finished, the TLC sheets were air-dried with nitrogen and analyzed under 254 and 365 nm UV light as well as chemically stained with Ehmann’s reagent (Ehmann 1977).

### HPLC analysis of *A. cinnamomea* extracts

The secondary metabolite profiling of *A. cinnamomea* extracts in acetonitrile was carried out on an HPLC 1100 system (Agilent Technologies, California, United States) with a reversed phase Eclipse Plus C18 USP L1 column (150 × 4.6 mm) under 254 nm UV detection. The injection volume was 20 μl. The mobile phase was a mixture of 0.87 mM H_3_PO_4_ (A) and acetonitrile (B). The gradient program was as follows: 10% B for 10 min; 10–50% B in 30 min; 50% B for 10 min; 50–100% B in 35 min; 100% B for 30 min. Flow rate was at 1 ml min^−1^.

### MS analysis

The MS analysis was carried out by a maXis™ ESI-QTOF mass spectrometer (Bruker Daltonics, Bremen, Germany). Samples were injected with direct infusion at 5 μl min^−1^. Mass spectrometer parameters were set as follows: capillary: 3500–3700 V, nebulizer: 10.2 psi, dry gas: 3 L min^−1^, dry temperature: 280 °C, collision energy: 10 eV, ion cooler RF: 550 Vpp, transfer time: 90 and 65 μs, pre puls storage: 30 μs, scan rate: 0.1 Hz, mass range: 50–3000 m/z. MS data were analyzed with DataAnalysis™ software (Bruker Daltonics).

## Results

### Growth of *A. cinnamomea*

**Fig. 1:**
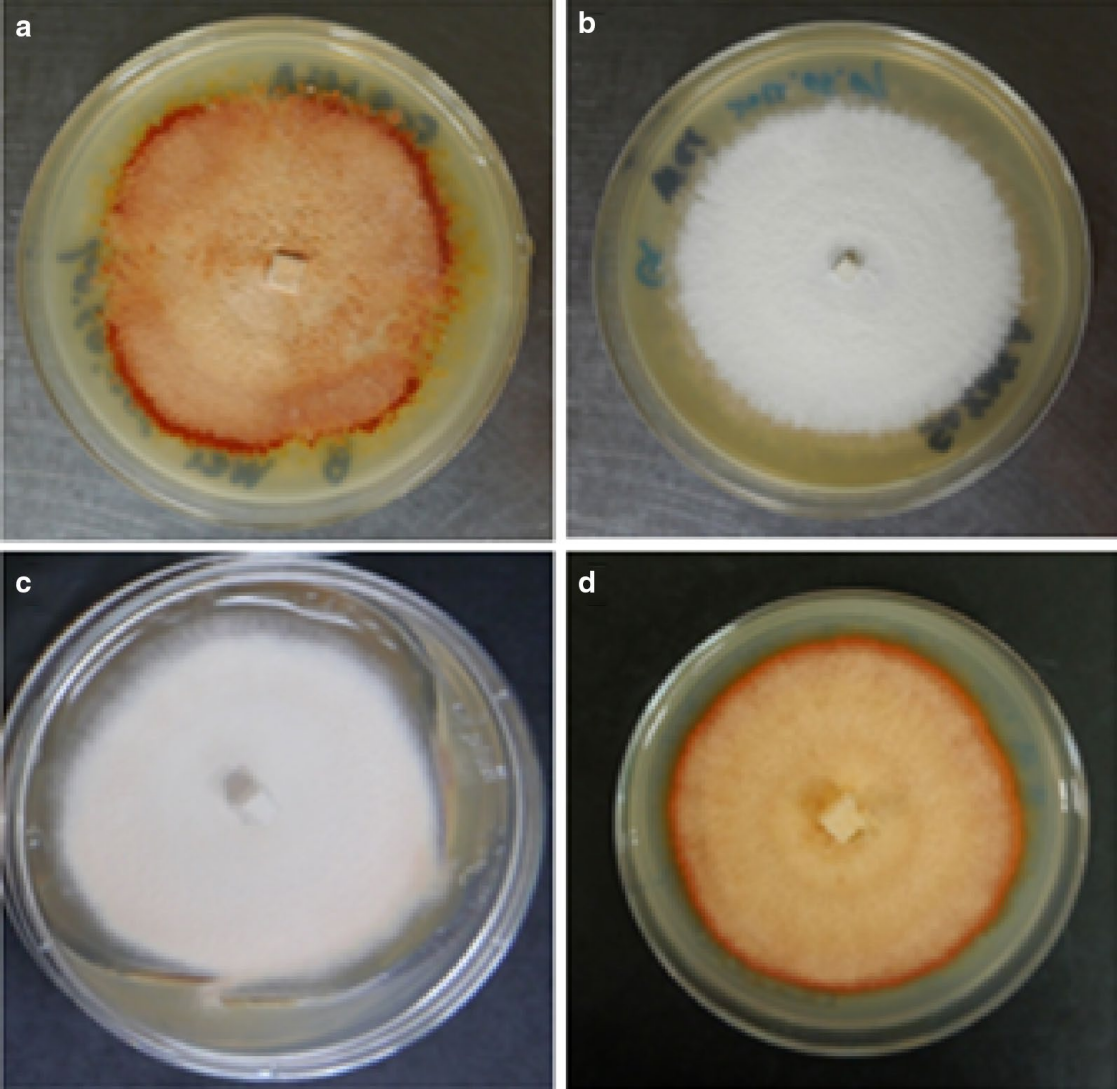
Morphology of 8-week old *Antrodia cinnamomea* on a 90 × 20 mm petri dish with 50 ml of 50% MEA media. **a** *A. cinnamomea* strain B under dark condition (regular); **b** *A. cinnamomea* strain G under dark condition (naturally occurring white); **c** *A. cinnamomea* strain B under continuous blue light radiation (induced white); **d** *A. cinnamomea* strain B under 6-week blue light radiation and following 2-week dark condition

**Fig. 2:**
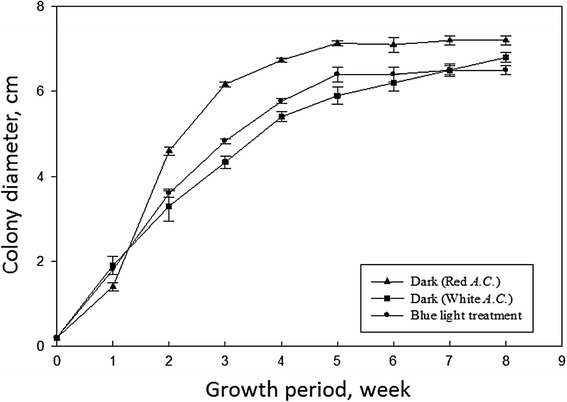
Growth curves of *Antrodia cinnamomea* with dark treatment of strains B (red *A. C.*) and G (white *A. C.*) and blue-light treatment of strain B

The morphology of 8-week-old colonies grown on 50% MEA media was shown in Fig. 1. The colony of strain B (Fig. 1a) denoted as regular form was red–orange. Its dark red margin indicated emerging fruiting bodies. Strain G was a naturally occurring white variant of *A. cinnamomea* (Fig. 1b), which did not form a fruiting body on 50% MEA. Strain B could be whitened when grown under continuous blue light radiation (Fig. 1c) but could be reversed by two-week growth in dark (Fig. 1d). The growth rates of *A. cinnamomea* on 50% MEA were shown in Fig. 2. Strain B in dark grew faster than the naturally occurring (strain G) and induced (strain B under blue light) white variants of *A. cinnamomea*.

### TLC analysis of *A. cinnamomea* ethyl acetate extracts

**Fig. 3:**
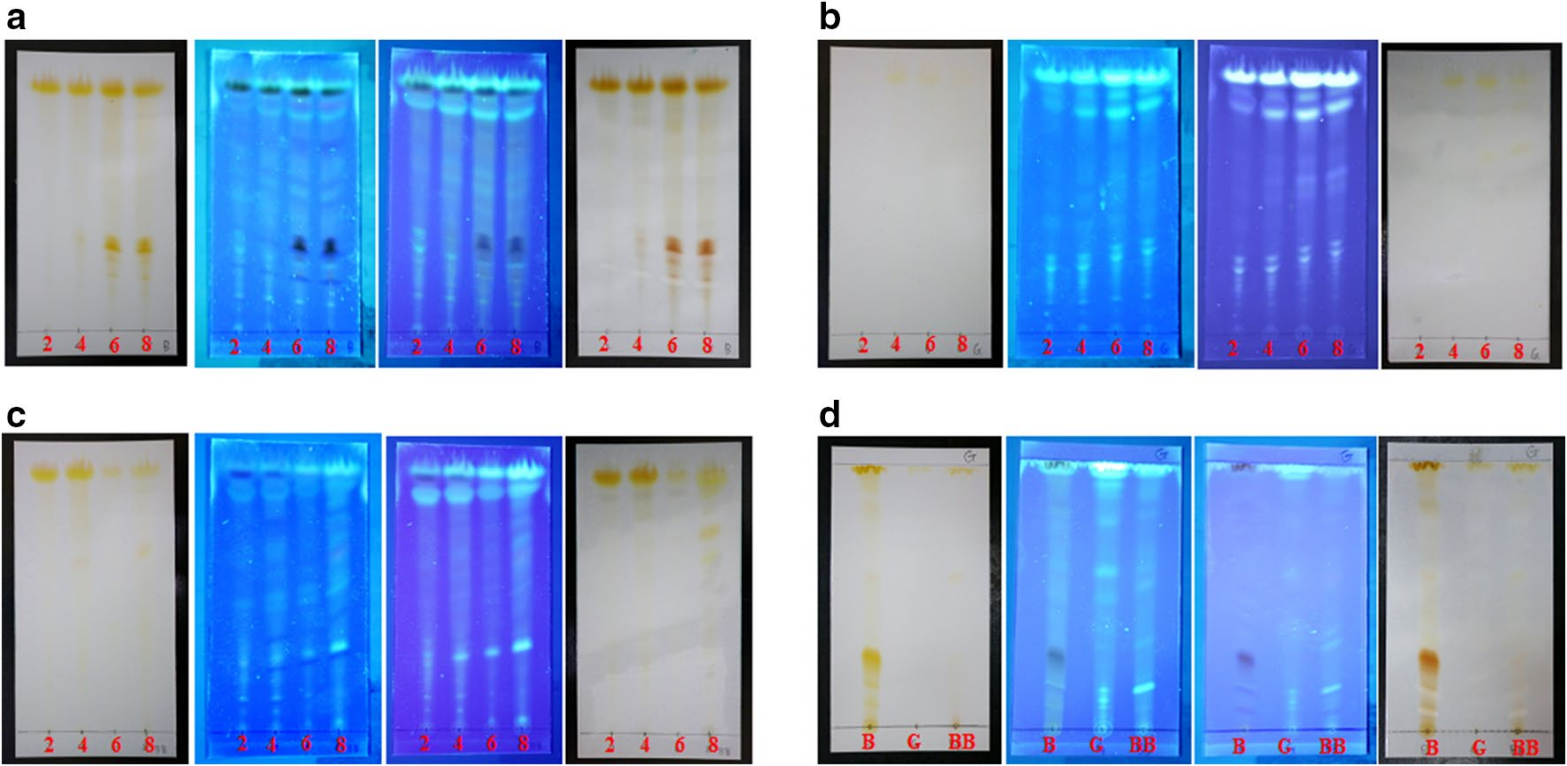
TLC analysis of ethyl acetate extracts of 2-, 4-, 6-, and 8-week old *Antrodia cinnamomea*. TLC plates were untreated, expressed under UV_254_, UV_365_, and Ehmann’s reagent staining from left to right. **a** regular *A. cinnamomea*; **b** naturally occurring white *A. cinnamomea*; **c** blue-light induced white *A. cinnamomea*. **d** TLC comparison of 8-week old *A. cinnamomea* strain B (B), strain G (G), and blue-light induced strain B (BB)

Colonies of *A. cinnamomea* at 2-, 4-, 6-, and 8-week old stages were collected and extracted with 100% ethyl acetate. The extracts were analyzed by TLC under UV_254_, UV_365_ and Ehmann’s reagent staining. The TLC profiles were shown in Fig. 3. Strain B under normal condition contained more complex chemical profiles (Fig. 3a, d), and strain G and the whitened strain B carried higher similarity in their chemical profiles (Fig. 3b–d). These results indicate some metabolic changes from the regular form to white variants of *A. cinnamomea* irrespective of naturally occurring or being induced.

### HPLC analysis and phenologic variation of major triterpenoids of *A. cinnamomea*

**Fig. 4:**
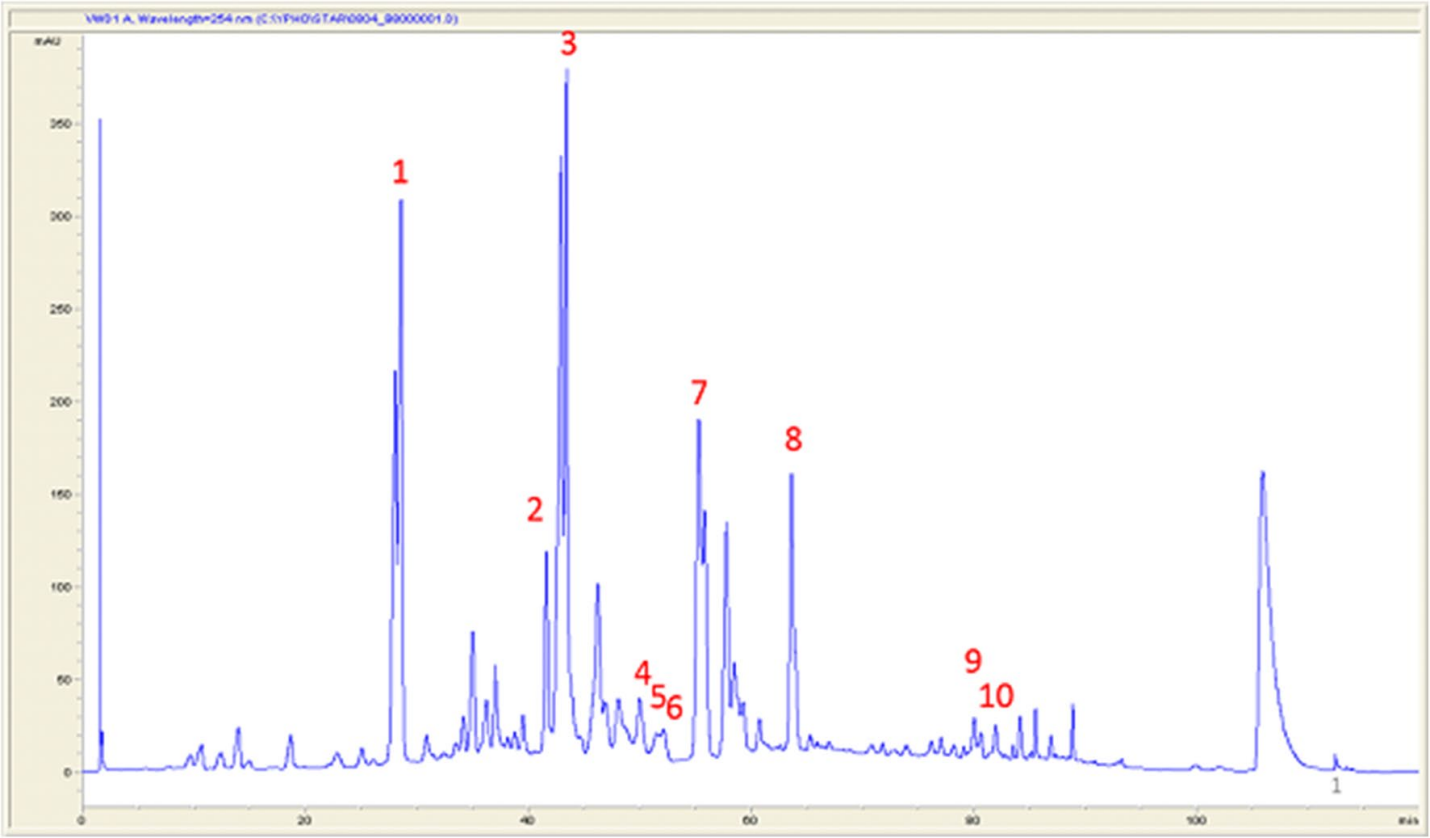
HPLC profile of ethyl acetate extract of 8-week old *Antrodia cinnamomea* strain B under dark condition showing 10 major triterpenoids. *1* Antcin K; *2* Antcin C; *3* Zhankuic acid C; *4* Dehydrosulphurenic acid; *5* Zhankuic acid B; *6* Sulphurenic acid; *7* Zhankuic acid A; *8* Antcin A; *9* Dehydroeburicoic acid; *10* Eburicoic acid

**Fig. 5:**
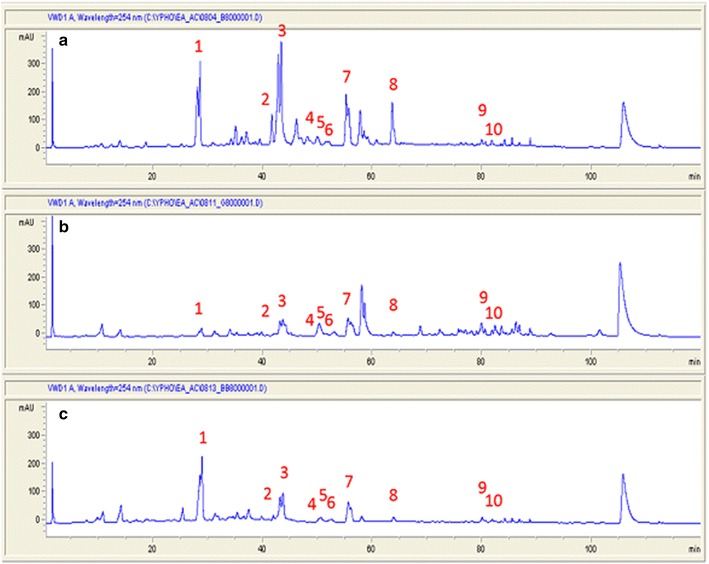
HPLC profile comparison of 8-week old *Antrodia cinnamomea* strains B (**a**), G (**b**) under dark condition and strain B under blue light radiation (**c**). Peaks numbered indicate the 10 major triterpenoids shown in Fig. 4

Ten major triterpenoids, including antcin A, antcin K, antcin C, dehydroeburicoic acid, dehydrosulphurenic acid, eburicoic acid, sulphurenic acid, zhankuic acid A, zhankuic acid B, and zhankuic acid C (Ao et al. 2009; Du et al. 2012; Huang et al. 2012), were identified and analyzed by HPLC and mass spectrometry. The HPLC profile of ethyl acetate extract from 8-week-old regular form of *A. cinnamomea* was shown in Fig. 4 with the ten major triterpenoids cited. The phenologic variations of these ten triterpenoids in the regular form (strain B under dark condition), the naturally occurring white variant (strain G), and the whitened variant (strain B under blue-light radiation) were shown in Tables 1, 2, 3, respectively. The HPLC profiles of the three 8-week old *A. cinnamomea* ethyl acetate extracts were compared in Fig. 5 indicating a significant difference between the regular form and white variants of *A. cinnamomea*.

**Table 1:** The weight percentages of ten major triterpenoids in the ethyl acetate extract of regular *Antrodia cinnamomea*

Triterpenoid compounds	Ages of regular *A. cinnamomea* (weeks)			
	2 (%)	4 (%)	6 (%)	8 (%)
Antcin K	2.436	4.601	6.893	6.808
Antcin C	1.426	1.837	2.288	2.703
Zhankuic acid C	1.754	4.743	7.697	9.342
Dehydrosulphurenic acid	0.54	1.315	1.504	1.636
Zhankuic acid B	0.868	2.043	0.815	0.643
Sulphurenic acid	–	–	1.504	1.636
Zhankuic acid A	5.599	4.274	4.369	5.254
Antcin A	1.844	4.044	4.622	4.447
Dehydroeburicoic acid	0.933	0.712	0.407	0.402
Eburicoic acid	0.529	0.35	0.674	0.513

**Table 2:** The weight percentages of ten major triterpenoids in the ethyl acetate extract of naturally occurring white *Antrodia cinnamomea*

Triterpenoid compounds	Ages of naturally occurring white *A. cinnamomea* (weeks)			
	2 (%)	4 (%)	6 (%)	8 (%)
Antcin K	0.317	0.107	0.72	1.105
Antcin C	0.41	0.286	0.495	0.412
Zhankuic acid C	0.523	1.073	2.124	2.16
Dehydrosulphurenic acid	0.935	1.623	2.445	3.408
Zhankuic acid B	–	–	0.46	0.465
Sulphurenic acid	–	0.265	1.189	1.076
Zhankuic acid A	3.152	3.366	5.011	2.695
Antcin A	0.261	0.487	0.648	0.614
Dehydroeburicoic acid	0.848	0.515	0.707	0.676
Eburicoic acid	0.566	0.833	1.021	0.83

**Table 3:** The weight percentages of ten major triterpenoids in the ethyl acetate extract of blue-light induced white *Antrodia cinnamomea*

Triterpenoid compounds	Ages of blue-light induced white *A. cinnamomea* (weeks)			
	2 (%)	4 (%)	6 (%)	8 (%)
Antcin K	1.277	3.604	11.348	10.987
Antcin C	0.169	0.142	0.349	0.912
Zhankuic acid C	1.27	6.722	3.684	5.629
Dehydrosulphurenic acid	0.999	0.774	1.551	1.563
Zhankuic acid B	0.864	0.502	0.511	1.234
Sulphurenic acid	0.382	0.73	–	–
Zhankuic acid A	5.323	6.753	1.205	4.095
Antcin A	1.247	2.317	0.248	1.027
Dehydroeburicoic acid	0.773	0.489	0.496	0.282
Eburicoic acid	0.235	0.362	0.959	0.681

## Discussion

In this study, we analyzed the chemical profiles of ethyl acetate extracts from a regular form, a natural occurring white variant, and a whitened variant induced by blue light at different growth stages. The TLC and HPLC analyses indicated that chemical profiles of ethyl acetate extracts from the regular form were getting more complex as the fungus grew while the white variants remained less complex (Fig. 3). This patterns became obvious when the regular form were 6 weeks old when the process of fructification started. This phenologic changes of the chemical profiles coincided with the morphological changes of the regular form from mycelium to fruiting body (Chu et al. 2010). There were no significant morphological changes in the naturally occurring white variant and the whitened variant. It indicates that their chemical profiles changed dramatically during fungal fructification and may produce novel medicinal effects.

A total of 39 triterpenoid compounds had been identified and structurally elucidated from *A. cinnamomea* based on the review of Geethangili and Tzeng (2011). These triterpenoid structures have an ergostane skeleton (Antcin A, C, K, Zhankuic acid A, B, and C in this study) or a lanostane skeleton (Sulphurenic acid, Dehydrosulphurenic acid, Eburicoic acid, and Dehydroeburicoic acid in this study) The ergostane-type triterpenoids are mostly found in fruiting bodies and rarely reported in mycelia when the triterpenoids of a wild *A. cinnamomea* fruiting body and those of a submerged culture were compared (Geethangili and Tzeng 2011). It is generally believed that ergostane-type triterpenoids are produced in fruiting bodies and lanostane-type triterpenoids exist both in fruiting bodies and in mycelia. However, this acknowledge of triterpenoid distribution in *A. cinnamomea* may not be true when *A. cinnamomea* was cultured in artificial agar-plate media. In our study, both ergostane-type and lanostane-type triterpenoids were detected in young mycelia and old mycelia with emerging of fruiting body. Most of the time, production of the ergostane-type triterpenoids was dramatically increased when the cultures were at 4–6 weeks old, but lanostane-type triterpenoids did not show a clear trend in quantity changes at this point (Tables 1, 2, 3). This indicates the biosynthesis of ergostane-type triterpenoids may be accelerated during *A. cinnamomea* fructification, but not lanostane-type triterpenoids. The lanostane-type triterpenoids may be involved in house-keeping during the growth and development of *A. cinnamomea*.

Differentiation and secondary metabolism are correlated processes in fungi that respond to light (Bayram et al. 2008). We analyzed ten key triterpenoids for their relative quantities during eight weeks of growth. In general, both naturally occurring white variant and induced whitened strains shared a higher pattern similarity than either with the regular strain. However, the blue-light induced whitened strain did contain a strong characteristic peak of antcin K, which was not shown much in the naturally occurring white strain (Fig. 5). This indicates the metabolism of triterpenoids in both the naturally occurring variant and the whitened strain may not carry the same pathways despite of both having white colonies. Antcin K which is considered a characteristic compound in *A. cinnamomea* fruiting bodies may be an early fructification-specific triterpenoid. Further fructification, which occurred in the regular strain, was seemingly blocked by blue-light treatment.

The effects of light treatment on fungal growth of various species had been studied earlier but with conflicted results. Chen and Dickman (2002) had shown that light treatment may induce a *Colletotrichum trifolii* TB3 kinase gene expression and help hyphal elongation and branching. However, the treatment of blue light radiation inhibited the apical growth of *Tuber borchii* mycelium by induction of a photoreceptor, Tbwc-1, gene expression (Ambra et al. 2004). Interestingly, in current studies, we found both phenomena occurred in our observation on growth of *A. cinnamomea*. The treatment of blue light radiation on regular *A. cinnamomea* increased the growth of fungal mycelium during the first ten days of inoculation, and then inhibited its growth thereafter (Fig. 2). This peculiar phenomenon indicates a more complex physiological interaction between fungal growth and light radiation. This phenomenon also suggests to us an improved procedure of culturing *A. cinnamomea* by treating cultures with light radiation for ten days before placing them in the dark.

## Conclusions

We found blue-light radiation could whiten the regular, orange-red *A. cinnamomea* by changing its expression of secondary metabolism and growth condition. Naturally occurring white variant did not show a better composition of triterpenoid profiles up to eight weeks old than that of regular strain. The ergostane-type triterpenoids were found existing both in young mycelia and old mycelia with fruiting body in artificial agar-plate medium culture, suggesting a more diversified biosynthetic pathway in artificial agar-plate culture rather than wild or submerged culture.

## Abbreviations

*A. cinnamomea*: *Antrodia cinnamomea*
*C. kanehirae*: *Cinnamomum kanehirae*
HPLC: high performance liquid chromatography
MEA: malt extract agar
MS: mass chromatography
TLC: thin layer chromatography
